# More Pronounced Bimanual Interference in Proximal Compared to Distal Effectors of the Upper Extremities

**DOI:** 10.3389/fpsyg.2020.544990

**Published:** 2020-10-27

**Authors:** Morten Andreas Aune, Håvard Lorås, Ane Djuvsland, Rolf Petter Ingvaldsen, Tore Kristian Aune

**Affiliations:** ^1^Department of Sport Science, Sport and Human Movement Science Research Group (SaHMS), Nord University, Levanger, Norway; ^2^Department of Teacher Education, Faculty of Social and Educational Sciences, Norwegian University of Science and Technology (NTNU), Trondheim, Norway

**Keywords:** bimanual coordination, bimanual interference, interhemispheric communication, movement constraints, upper-limb coordination

## Abstract

Bimanual performance depends on effective and modular bilateral communication between the two bodysides. Bilateral neural interactions between the bodysides could cause bimanual interference, and the neuromuscular system for proximal and distal muscles is differently organized, where proximal muscles have more bilateral interneurons at both cortical and spinal level compared to distal muscles. These differences might increase the potential for bimanual interference between proximal arm muscles, because of greater proportions of bilateral interneurons to proximal muscles. The purpose of the present experiment was to evaluate potential differences in bimanual interference between proximal versus distal effectors in the upper extremities. 14 participants first performed a unilateral primary motor task with dominant arm with (1) a proximal and (2) distal controlled joysticks (condition A). Performance in condition A, was compared with the same effector’s performance when a bimanual interference task was performed simultaneously with the non-dominant arm (condition B). The results showed a significant bimanual interference for both the proximal and distal controlled joysticks. Most interestingly, the bimanual interference was larger for the proximal joystick compared to the distal controlled joystick. The increase in spatial accuracy error was higher for the proximal controlled joystick, compared with the distal controlled joystick. These results indicate that the proximal-distal distinction is an important organismic constraint on motor control, and especially for bilateral communication. There seem to be an undesired bilateral interference for both proximal and distal muscles. The interference is higher in the case of proximal effectors compared distal effectors, and the results seem to map the neuroanatomical and neurophysiological differences for these effectors.

## Public Significance Statement

From a professional perspective it is important to find solutions to overcome and reduce bimanual spatial and temporal interference during control of planes, drones, surgery and so on, and the present study focus on man-machine interface and how the use of proximal compared to distal joysticks controlled by the dominant arm deals with bimanual interference when moving the non-dominant arm simultaneously. The performance with both the proximal and distal controlled joystick’s controlled with the dominant arm (the primary task) is negatively affected by the introduction of a bimanual interference task performed with the non-dominant arm, but most interestingly, the bimanual interference was larger for the proximal joystick compared to the distal controlled joystick. It is likely to recommend distal controlled joysticks in order to resist bimanual motor perturbations.

## Introduction

In many everyday motor tasks and sport specific motor skills, success depends on the degree of collaboration between the two arms and hands (Swinnen and Gooijers, 2015; Hesse et al., 2018; Nemani et al., 2018). In particular, the Western (modern) way of life with its increasing complexity, requires frequently the need to use both hands simultaneously in order to operate and control various devices. Whether the arms and hands need to work together or perform different operations depends on the task constraints. Increased knowledge about potential neuromuscular constraints caused by the neuroanatomical and neurophysiological architecture and its effect on motor control and coordination when performing bimanual tasks is thus interesting for understanding motor performance in many motor activities in both the professional and recreational life (Swinnen and Gooijers, 2015; Hesse et al., 2018). For example, dribbling in basketball, playing piano, and in many man-machine interfaces such as controlling airplanes, drones and performing robotic surgery, require a certain degree of temporal and spatial dissociation while simultaneously moving arms and hands. It is well-known that the human capacity for multi-tasking is quite limited (Abernethy, 1988; Pashler, 1994; Tombu and Jolicoeur, 2003; Kolb and Whishaw, 2009; Schott and Klotzbier, 2018) and disentangling the precise mechanisms that might facilitate or hinder bimanual task performances might help to identify the most effective strategies for overcoming potential spatial and temporal interference during the completion of such tasks.

Performance and control of bimanual tasks is highly modular, which enables the fulfilment of different task requirements. For bimanual control and coordination, the two body sides corticospinal tracts can to some extent be separated and recombined (to a certain degree), giving greater flexibility and variety in motor performance (Levin et al., 2004; Swinnen and Wenderoth, 2004). Real-life bimanual motor tasks that require isomorphic (similar) actions by both sides is for example pushing a box, lifting heavy objects or rowing, whereas others motor tasks might require unimanual motor control of left and right body sides and play a more differentiated role, such as sewing, driving a car, opening a bottle or playing different instruments and so on (Swinnen, 2002; Swinnen and Wenderoth, 2004). Furthermore, performance of many bimanual tasks requires the control and coordination of a bewildering complexity of operations in a sophisticated manner, whereas other types of bimanual task have relatively few solutions (Swinnen, 2002).

The role of bimanual control and coordination in various motor skills has interested researchers for many decades (Kelso, 1984; Pashler, 1994; Wenderoth et al., 2003; Kolb and Whishaw, 2009), and understanding the contribution of such control and coordination processes in complex motor skills is a critical objective for both cognitive and neurophysiological researchers (Swinnen and Gooijers, 2015). In addition, from a practical point of view, it is considered an important entry point for studies of potential bimanual interference when the two arms are operated simultaneously. The results obtained from several studies suggest that control and coordination of bimanual tasks is compromised when two tasks with different spatial and/or temporal trajectories have to be performed simultaneously (Sherwood, 1994; Spijkers and Heuer, 1995; Cattaert et al., 1999; Heuer et al., 2001; Swinnen et al., 2001; Wenderoth et al., 2003; Aune et al., 2013). For example, bimanual interference emerges when two limbs must be moved along different trajectories and conducted under different task parameters (Wenderoth et al., 2003). The main parameters affecting bimanual interference appear to be the amplitude and direction of movement, along with velocity and shape assimilation (Franz, 1997; Walter et al., 2001; Weigelt et al., 2007; Calvin et al., 2010).

Research into bimanual movements is important from a cognitive perspective, because higher cognitive functions are involved in complex movements that require perception and action, as well as executive functions such as task-switching (Swinnen and Wenderoth, 2004; Swinnen and Gooijers, 2015). In terms of information-processing perspectives, bimanual movements are considered to cause interference between a main task and subtasks that have to be performed simultaneously, due to limitations on neural resources (Marteniuk et al., 1984; Swinnen et al., 1991; Cattaert et al., 1999; Heuer et al., 2001; Diedrichsen et al., 2003; Swinnen and Gooijers, 2015). Furthermore, these limitations in neural resources during information-processing and motor programming are likely to cause bimanual interference for execution of bimanual movements (Swinnen and Wenderoth, 2004; Hesse et al., 2018).

In neurophysiological terms, the corpus callosum and interneurons in the spinal cord probably have a prominent role in the bilateral communication required for the execution of bimanual tasks (Jeeves et al., 1988; Heuer et al., 1995; Swinnen, 2002; Wenderoth et al., 2003; Swinnen and Gooijers, 2015). Bilateral neural interactions are essential for transfer and integration of information between hemispheres and corticospinal tracts. It has been suggested that the bilateral communication can be inhibitory and decrease neural drive to the contralateral muscles during some bilateral motor actions (Delwaide and Pepin, 1991; Ferbert et al., 1992; Bannatyne et al., 2003; Hübers et al., 2008; Takeuchi et al., 2012), but interhemispheric interactions can also be excitatory and increase neural drive to the contralateral muscles during bilateral motor actions (Serrien and Brown, 2002; Bloom and Hynd, 2005; Bannatyne et al., 2006; Liuzzi et al., 2011).

Several studies have shown that neural drive to the contralateral hemisphere and muscles can both increase and decrease when performing both unilateral and bimanual motor actions and muscle contractions, and it is suggested that the activation probably depend on the type of movement and purpose of the motor actions (Oda and Moritani, 1995; Taniguchi et al., 2001; Schultze et al., 2002; Khodiguian et al., 2003). The number of transcallosal projections (commissural fibers through corpus callosum) and bilateral interneurons in the spinal cord connecting proximal muscles are higher compared to distal muscles in primates (Pandya and Vignolo, 1971; Jenny, 1979; Gould et al., 1986; Rouiller et al., 1994; Brodal, 2004), and it probably increase the potential for bilateral interference. It is also interesting that the corticospinal projections for distal arm muscles are mainly direct through monosynaptic connections, while the corticospinal projections for proximal arm muscles are polysynaptic (Kuypers, 1978; Palmer and Ashby, 1992). It is suggested as a consequence of the greater proportion of monosynaptic connections between motor cortex and distal muscles that it might weaken the potential for interference for those muscles.

Such neuroanatomical and neurophysiological differences between proximal and distal muscles might impact the potential for bilateral communication and bilateral interference for these effectors, which in some tasks might be an advantage, while in other tasks be a disadvantage. The significantly higher number of commissural axons for proximal muscles probably increases bilateral communication (both inhibitory and excitatory) with these effectors (Ferbert et al., 1992; Bannatyne et al., 2003; Bloom and Hynd, 2005), and it is hypothesized to cause a more pronounced bilateral interference in bimanual motor actions for effectors compared to distal effectors.

The purpose of the present study was to test this hypothesis by investigating potential differences in bilateral interference between proximal and distal effectors in the upper extremities by perturbing with a bimanual coordination task. Specifically, by comparing the extent to which performance of a proximal joystick versus distal joystick controlled with the dominant arm is affected by a bilateral task performed simultaneously with the non-dominant arm. If bimanual interferences are associated with functional and structural differences in the motor system for proximal versus distal effectors, there should be more pronounced bimanual interference for the proximal controlled joystick compared to the distal controlled joystick.

## Materials and Methods

### Participants

The sample comprised 14 university students with no known neuromuscular disorders or functional limitations (including normal and corrected vision) were recruited, seven women (mean age 23.9, SD = 7.5 years) and seven men (mean age 25.1, SD = 1.6 years). Based upon baseline (pre-test) proximal-distal differences in absolute spatial error (ASE) in a similar experimental task from a previous study (Aune et al., 2017), it was estimated that this sample size was sufficient to achieve a power of 80%, a level of significance of 5% (two sided) and effect size (Cohen’s *d*) at 0.9, for detecting a mean proximal-distal differences in ASE that are significant different from zero (G^∗^Power: Faul et al., 2007). All participants were right-handed as indicated by the Edinburgh Handedness Inventory (Oldfield, 1971) with a mean laterality index score of 0.94 (SD = 0.06). None of the participants reported any specialized training of the upper extremities through occupation and sports (e.g., strength training, playing piano). All participants gave informed consent prior to the experimental procedure. The study was evaluated and approved by the Regional Ethical Committee and performed in accordance with the Declaration of Helsinki.

### Task and Apparatus

To compare bimanual interference in proximal versus distal effector systems the participants first performed a unilateral primary motor task with their dominant arm with both the proximal (shoulder and elbow) and distal (wrist and fingers) effector systems (condition A). Performance in condition A, using both effector systems, was compared with the same effectors performance when a bimanual interference task had to be performed simultaneously with the non-dominant arm (condition B).

#### Primary Task

**FIGURE 1:**
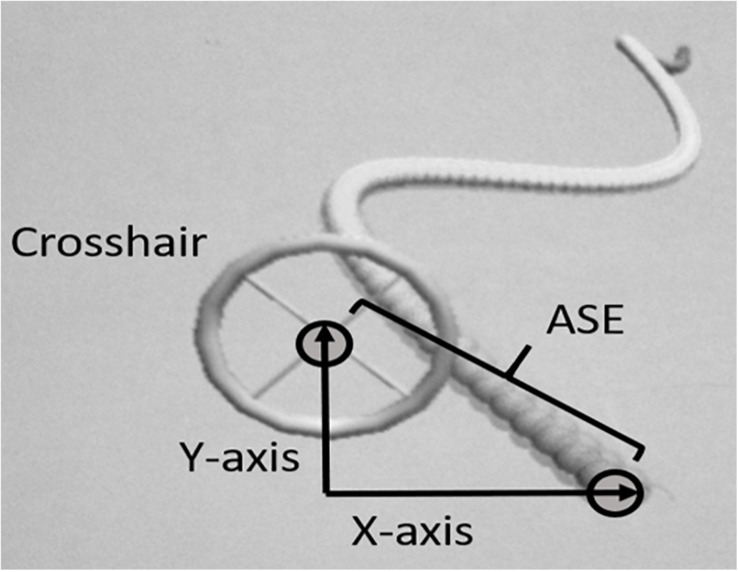
Illustration of the customized 2D virtual snake task and calculation of the absolute spatial error (ASE).

The primary task used in the present study is the same task as we used in an earlier published study of learning transfer using proximal and distal joysticks (Aune et al., 2017). The primary motor task was a specially designed version of a 2D virtual “moving snake” task. The moving snake consisted of a two-dimensional (x and y) complex periodic waveform made by the head of the snake and participants had to use a controllable crosshair to track the target (head of the snake) as precisely as possible (see Figure 1). The diameter of the head of the snake and the center of the crosshair as you see it on the on the screen is 50 and 30 mm, respectively. The criterion waveform was the same in every trial. In both conditions the participants were instructed to position the center of the crosshair at the head of the snake and follow the undulating movement of the snake’s head as closely as possible. When the center of the crosshair was perfectly positioned on the head of the snake (identical x and y positions for the crosshair and the head of the snake) the color of the snake’s head changed, thus providing online feedback to the participant. The moving snake task was designed using the Unity3D game engine and programmed using C#. The sampling frequency used for the task was 100 Hz. At each sampling point the following information was stored: time from the start of the game; x- and y- coordinates of target; x- and y-coordinates of crosshair.

**FIGURE 2:**
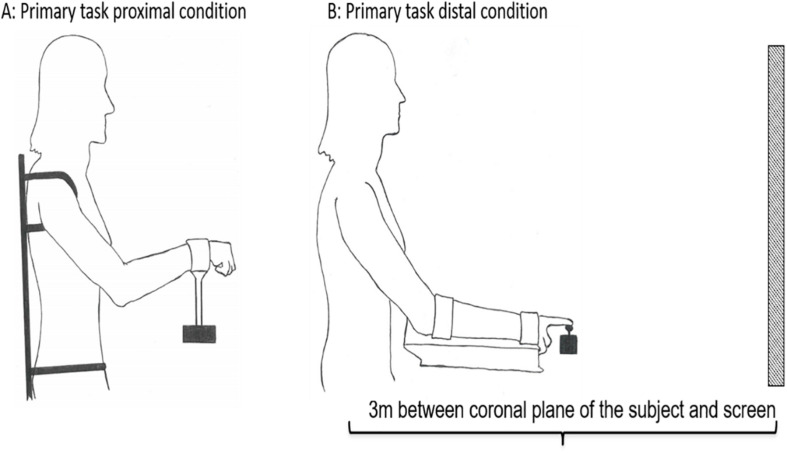
Experimental set-up of the primary task. The subject was positioned seating 3 m from the screen in both conditions. In order to prevent mechanical, postural, and synergist muscle contributions in the proximal **(A)** and distal **(B)** conditions, the participants’ body positions were constrained by clamps and straps as illustrated. The starting position in the proximal condition was calibrated to 45° between the trunk and overarm (humerus), and 130° between humerus and radius **(A)**. The starting position in the distal condition was calibrated to 25° between the trunk and overarm, with the underarm resting in a horizontal position **(B)**.

To perform the primary task the participants used two different customized joysticks to perform isolated unilateral movements of the proximal and distal effectors: (1) a customized proximal joystick controlled by shoulder and elbow, and (2) a customized distal joystick controlled by wrist and index finger. The movement of both the proximal and distal joysticks were a combination of flexion-extension and adduction-abduction, and had a maximum angular amplitude (maximum range of movement) set to 30° in each direction from the neutral starting position of the two joysticks. The joysticks were operated unilaterally, with the dominant side, in both conditions. The participants were positioned in a chair 3 m from the screen. A custom-made chair and apparatus were used to prevent postural instability and activation of other postural core muscles, thereby limiting activation to the shoulder-elbow in the proximal condition and the wrist-index finger in the distal condition. In the proximal condition the trunk and upper body were strapped to the chair to limit movement to the shoulder and elbow (see Figure 2) and the seat was elevated to eliminate activation of the feet. In the distal condition the wrist and index fingers were isolated by strapping the forearm to a platform (see Figure 2). The screen had a digital resolution of 60 Hz (1920 × 1080) and a width of 148 cm and a height of 110 cm. The amplitude of the “moving snake” was adjusted to be approximately 1/3 of the height of the screen.

#### Bimanual Interference Task

**FIGURE 3:**
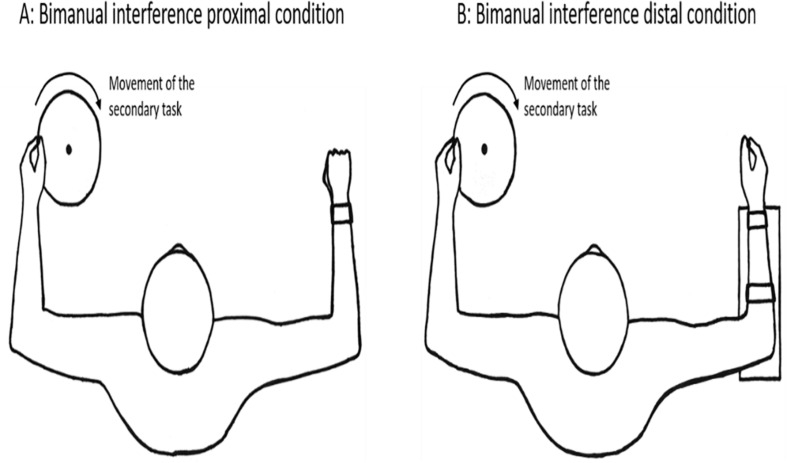
Experimental set-up of the bimanual interference task for proximal versus distal test conditions.

The bimanual interference task performed simultaneously as the primary task, was a constrained circular motion performed with the non-dominant arm consisting of rotating a disk (diameter = 21 cm) that required activation of both proximal (shoulder and elbow) and distal (wrist and fingers) effectors (see Figure 3). The participants held the rotating disk by gripping a small bar that was attached to the disk with their index finger and thumb (pincer grip). They were instructed to rotate the disk with an inward rotation direction (clockwise for right handed participants) continuously at a steady speed of about 1 Hz.

### Procedure

The experiment was completed over two consecutive days. The participants were naïve to the hypotheses of the study. On the first day, the participants were informed about the task and demonstration of it by the experimenter. Then the participants completed a training session of the two primary tasks in the following conditions: (1) Proximal joystick controlled with dominant arm, and (2) Distal joystick controlled with dominant arm. The participants completed 20 practice trials with both the proximal and distal joysticks in order to familiarize themselves with the experimental set-up, the primary task, and the two different joysticks. Each training session was subdivided in four blocks of five trials each, followed by a 2 min rest period, in order to maintain motivation and prevent fatigue. At the end of each training session the experimenter provided verbal feedback, accompanied by visual graphic feedback of the last trial on the screen in front of the participants. The order of proximal and distal conditions in the training session was counterbalanced across participants.

On the second day the experiment followed an AB-design. In condition A, performance of the primary task was tested with both the proximal and distal joysticks. All participants performed one practice trial of the unilateral primary task using the proximal and distal joysticks before being tested in condition A. In condition A, the dependent variable consisted of a primary task controlled with both (1) a distal joystick and (2) a proximal joystick unilaterally, and with no bimanual interference task introduced. In total three trials were performed with both the proximal and distal joysticks, in total six testing trials in condition A. Condition A provided baseline performance of the primary task in the absence of interference. This was followed by condition B, in which the participants had to perform the bimanual interference task simultaneously as the primary task. In condition B, the bimanual interference task (rotating a disk with a circular motion with the non-dominant arm) was introduced. The participants had to perform a bimanual coordination task, i.e., to perform the primary task with their dominant arm, whilst simultaneously performing the bimanual interference task with the non-dominant. The participants were instructed to focus on maintaining performance of the primary task, and clearly have second priority on the bimanual interference task. Also in condition B three trials were performed with both the proximal and distal joysticks, in total six testing trials. The order in which the two levels of the independent variable (proximal versus distal controlled joysticks) were tested, was counterbalanced across participants to eliminate potential learning effects related to operation of the proximal and distal joysticks in conditions A and B. The primary task was performed in the same order in both condition A and condition B.

### Data Analysis

The analysis of the primary task used in the present study is the same analysis as we used in an earlier published study of learning transfer using proximal and distal joysticks (Aune et al., 2017). The dependent variable was measured as the average ASE in positioning of the crosshair relative to the target. The unit of measurement was virtual meters (VM) as defined in the customized software. ASE was measured as the distance between the head of the snake and the middle of the crosshair, calculated using Pythagoras equation (see Figure 1):

$$\mathrm{Absolute}\mathrm{Spatial}\mathrm{Error}\left(\mathrm{ASE}\right)=\sqrt{(x^{2}}+y^{2})$$

Performance in all conditions was tested over a 50 s period in each trial with a sampling frequency of 100 Hz, which amounts to a total 5000 samples per trial. In total three trials were performed in each condition. In order toanalyze steady state performance only samples from 300 to 4700 were analyzed, a total 4400 samples per condition. The first and the last 300 samples were excluded because performance in the early part of the test period might have been influenced by tuning in to the experimental task, whereas the final 300 samples were excluded to avoid effects due to loss of concentration, fatigue, or mobilizing extra effort (e.g., Repp and Penel, 2004; Moe-Nilssen and Helbostad, 2005; Lorås et al., 2012). The time for each trial was displayed at the screen shown as a countdown of the duration of the test. Average ASE was calculated across three trials and used in subsequent analyses. The change in movement accuracy (ΔASE) between *no bimanual interference* and *with bimanual interference* were used to describe the interference effects for proximal versus distal joysticks.

Video analyses of the bimanual interference task were used to confirm that the participants were moving in accordance with the instructions. Trials that deviated temporally more than ±0.1 Hz were eliminated from further analyses (in total <5%).

### Statistical Analysis

Shapiro–Wilk tests indicated that all variables were normally distributed. Thus, the effect of bimanual interference on control of the proximal and distal joysticks was examined with a 2 (proximal or distal effector) × 2 (condition A or B) within-subject repeated measures ANOVA on the ASE. In the repeated measures ANOVA, partial eta squared (η^2^*p*) was applied as the indicator of the effect size interpreted as small effect: 0.01, medium effect: 0.06, and large effect: 0.14 (Cohen, 1988; Richardson, 2011). *Post hoc* Bonferroni corrected pairwise comparisons at the level of simple main effects on accuracy (ΔASE) were conducted with paired samples *t*-tests: proximal effector (no interference vs. interference), distal effector (no interference vs. interference), no interference (proximal vs. distal) and with interference (proximal vs. distal). Furthermore, a paired samples *t*-test was applied to compare the increase in ASE (from no interference vs. with interference) between proximal and distal effectors. For dependent *t*-tests, Cohen’s *d*_*Z*_ was applied as a measure of the effect size (Lakens, 2013), in which 0.2, 0.5, and 0.8 were considered small, moderate, and large, respectively (Cohen, 1988). Calculation of CI on effect size point estimates (partial eta^2^, Cohen’s *d*_*Z*_) were conducted by syntax provided by Professor Karl Wuensch^1^. Predictive Analytics Software (PASW, IBM, United States; previously SPSS) Version 26.0 was applied for statistical calculations, with alpha = 0.05 as criterion for statistical significance.

## Results

**FIGURE 4:**
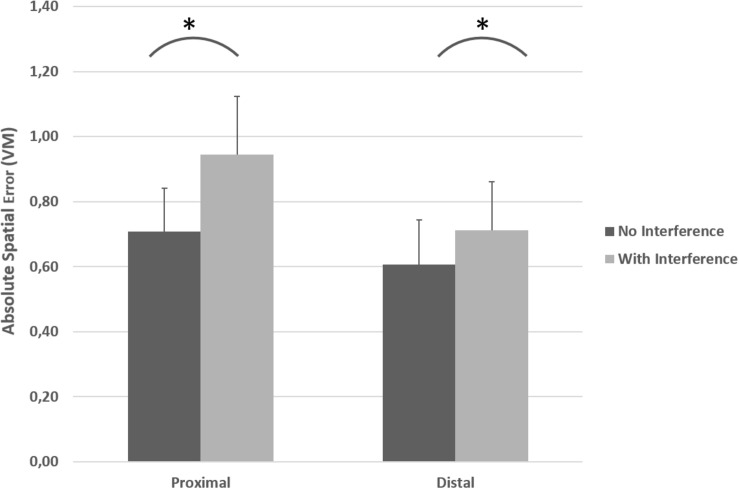
Absolute spatial error (ASE) exerted for both the unilateral primary task (condition A: black bars) and with the introduction of the bimanual interference task (condition B: gray bars) for the proximal and the distal joysticks. * indicates significant increase in ASE in the primary task because of the bimanual interference task performed simultaneously. The error bars illustrate SD.

As depicted in Figure 4, ASE was higher for proximal joystick-control compared to distal joystick-control in both condition A (without interference) and condition B (with bimanual interference). Also visible in Figure 4, the introduction of bimanual interference increased ASE in both proximal and distal conditions. A repeated measures ANOVA indicated a significant effector (proximal or distal) × condition (A or B) interaction effect on ASE [*F*(1, 13) = 18.63 *p* = 0.001, η^2^*p* = 0.59 (90% CI [0.23; 0.73])]. Repeated measures ANOVA indicated a main effect of proximal vs. distal effector on ASE [*F*(1, 13) = 33.41, *p* < 0.001, η^2^*p* = 0.72 (90% CI [0.41; 0.82])] with a mean difference at 0.17 VM (95% CI [0.10; 0.23]) and a significant main effect of condition (with or without interference) on ASE [*F*(1, 13) = 50.59, *p* < 0.001, η^2^*p* = 0.80 (90% CI [0.54; 0.87])] with a mean difference at 0.17 VM (95% CI [0.12; 0.22]).

**FIGURE 5:**
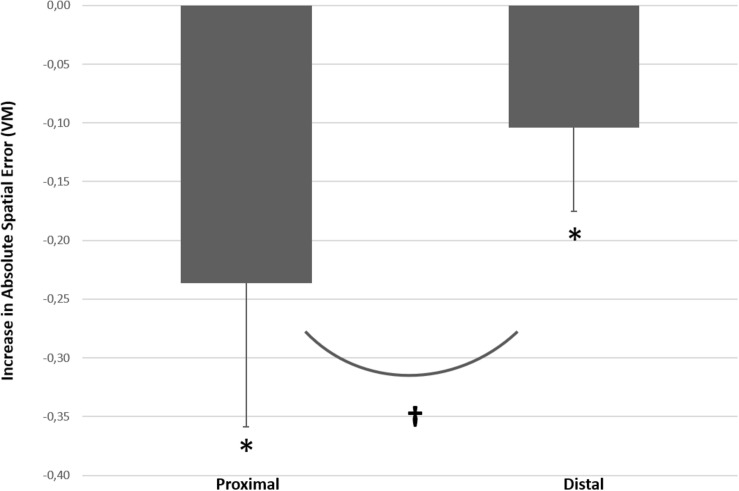
Increase absolute spatial error (ΔASE) between *no bimanual interference* and *with bimanual interference* (the interference effects) for proximal vs. distal joysticks. * indicates that ΔASE Index score is significantly different from zero for both proximal and distal joysticks respectively, and † indicates significant difference in ΔASE between proximal effectors and distal effectors with the introduction of the bimanual interference task. The error bars illustrate SD.

*Post hoc* analysis with paired *t*-tests indicated at the level of proximal effector, significant higher ASE (mean difference = 0.23, 95% CI [0.17; 0.30]) *with* interference compared to *no* interference [*t*(13) = 7.23, *p* < 0.001, *d*_*Z*_ = 1.93 (95% CI [1.02; 2.82])]. Similarly, at the level of distal effector, ASE was significantly higher (mean difference = 0.10, 95% CI [0.06; 0.14]) *with* interference compared to *no* interference [*t*(13) = 5.50, *p* < 0.001, *d*_*Z*_ = 1.47 (95% CI [0.69; 2.22])]. Furthermore, at the level of *no* interference ASE was higher (mean difference = 0.10, 95% CI [0.02; 0.05]) with proximal effector compared to distal effector [*t*(13) = 4.16, *p* = 0.001, *d*_*Z*_ = 1.11 (95% CI [0.42; 1.77])]. In conditions *with* interference, ASE was also higher (mean difference = 0.23, 95% CI [0.15; 0.32]) in proximal compared to distal effector [*t*(13) = 5.19, *p* < 0.001, *d*_*Z*_ = 1.39 (95% CI [0.63; 2.11])]. As can be seen in Figure 5, the increase in ASE (from no interference vs. with interference) was significantly larger for proximal controlled joystick compared to distal controlled joystick [*t*(13) = 4.31, *p* = 0.001, *d*_*Z*_ = 1.15 (95% CI [0.46; 1.82])].

## Discussion

The current study investigated bimanual interference in the upper extremities by comparing performance of a unilateral motor task (primary task only; condition A) with a bimanual interference task performed simultaneously (condition B). The results indicated that control of a joystick with distal effectors was more precise (lower ASE) compared to the joystick controlled by proximal effectors (see Figure 4). Furthermore, bilateral motor interference (a decrease in spatial accuracy on the primary task in the bimanual condition) occurred with both proximal and distal controlled joysticks. However, bimanual interference was more pronounced with the proximal controlled joystick compared to the distal controlled joystick; the average increase in ASE was 0.246 VM with proximal controlled joystick, compared with 0.104 VM with the distal controlled joystick (see Figure 5).

These findings indicate that distal effectors provide better joystick control compared to proximal effectors. The proximal-distal gradient observed in this study is consistent with the results of other studies from our laboratory, which have demonstrated a proximal-distal gradient for bilateral transfer of learning (Aune et al., 2017) and bilateral force deficits (Aune et al., 2013). These behavioral studies demonstrate that the proximal-distal distinction is an important organismic constraint on human motor control. The behavioral data seem to map on to the neuroanatomical and neurophysiological differences between proximal and distal effectors. Performing a bimanual interference task simultaneously with the execution of the primary task, likely activate contralateral motor neurons, and subsequently interfere with motor commands for the primary task (Ferbert et al., 1992; Bannatyne et al., 2003; Bloom and Hynd, 2005). The potential for bimanual interference is thus higher in the case of proximal effectors compared distal effectors, possibly because of their different neuroanatomical and neurophysiological organization, and might explain the proximal-distal gradient observed for the upper extremities.

Motor task performance, measured as ASE in the current study, was found to be worse with both proximal and distal joystick control when participants also had to generate simple circular motions with their non-dominant hand (see Figure 4). A general performance decrement in one or both tasks is a common finding in bimanual coordination tasks (condition B in this study) (Ebersbach et al., 1995; Baars and Gage, 2010; Magill and Anderson, 2017). This implies that, in bimanual motor tasks where both hands must be separately controlled, the degree of bilateral modularity in nervous signals probably is higher, in contrast to more bilateral isomorphic actions (Swinnen and Wenderoth, 2004).

The general decrease in spatial accuracy observed in both proximal and distal conditions of the current study clearly suggest that bimanual coordination causes interference between the main task (joystick control) and the bimanual interference task involving a movement (circular motions) that is performed simultaneously. At a cognitive level, these findings can be explained by a higher amount of information processing during motor programming and execution of bimanual movements, and by limitations in neural resources (Swinnen et al., 1991; Cattaert et al., 1999; Heuer et al., 2001; Swinnen and Wenderoth, 2004; Swinnen and Gooijers, 2015; Hesse et al., 2018). This effect is attributed to the neuroanatomical and neurophysiological system’s limited capacity to carry out two tasks simultaneously (Klingberg and Roland, 1997; Cockburn, 1998; Swinnen and Wenderoth, 2004), because neural resources must be shared–at least to some extent–between information processing and execution (Abernethy, 1988; Pashler, 1994; Kolb and Whishaw, 2009; Magill and Anderson, 2017).

As hypothesized, the degree of bimanual interference was higher for the proximal controlled joystick compared with the distal controlled joystick (see Figure 5). The more pronounced bilateral interference with the proximally controlled joystick might be explained by the difference in the number of commissural interneurons that cross the midline and interact with contralateral motor neurons during performance of a bimanual motor task.

There is a difference for proximal compared distal muscles in potential for bilateral interaction at a higher order in the nervous system (at both the cortical and spinal level). The number of commissural fibers in corpus callosum is higher for proximal compared to distal muscles (Pandya and Vignolo, 1971; Brinkman and Kuypers, 1972; Jenny, 1979; Gould et al., 1986; Aglioti et al., 1993; Brodal, 2004; Jankowska et al., 2005b; Kolb and Whishaw, 2009), and in addition, there are more bilateral interneurons in the spinal cord for proximal compared to distal muscles. Hence, the bilateral interaction and communication between the two hemispheres and body sides is relative greater for proximal compared to distal muscles (Jeeves et al., 1988; Heuer et al., 1995; Swinnen, 2002; Wenderoth et al., 2003; Swinnen and Gooijers, 2015), and thereby might explain the more pronounced bimanual interference for proximal compared to distal joysticks.

At the cortical level, differences in bimanual interference between proximal and distal muscles with respect to bimanual interference arise due to differences in interhemispheric communication, which is highly important for efficient coordination of bimanual motor tasks (Gooijers and Swinnen, 2014). Several studies have suggested that excitatory and inhibitory interhemispheric communication via the corpus callosum is required to perform bimanual tasks (Kinsbourne, 1975; Lassonde, 1986; Hellige, 1993; Bloom and Hynd, 2005; Takeuchi et al., 2012). In bimanual motor control, the corpus callosum interacts bilaterally with the primary motor cortex (Bloom and Hynd, 2005), and the motor system needs to activate both hemispheres simultaneously. The higher number of commissural fibers related to proximal effectors in the corpus callosum may cause a greater potential of bilateral communication to proximal muscles, and then might manifest as increased bimanual interference, as observed in proximally controlled joystick condition in this study. Furthermore, there are more commissural interneurons related to proximal compared to distal effectors in the spinal cord, and this is reflected in bilateral control of muscles (Jankowska, 1992; Pierrot-Deseilligny and Burke, 2005). The contralateral spinal circuits might generate a gain in modulation through commissural interneurons (Hortobágyi et al., 2003), which in turn would result in contralateral motor neurons receiving enhanced excitatory or inhibitory nervous signals (Jankowska et al., 2005a, b) and cause bilateral interference. In addition, people might be more familiar using fingers and wrist to manipulate and control objects rather than using the shoulder and elbow. Hence, more cognitive resources (e.g., motor planning) might be required to perform such an unexperienced task when controlling the proximal joystick, and it might impose a higher cognitive load that potentially can be associated with the observed higher ASE in both condition A (no interference) and B (with interference). Therefore, it would be interesting in future studies to evaluate whether the observed differences (proximal-distal distinction) disappear or become smaller with motor practice or bimanual expertise.

### Limitations and Future Perspectives

The behavioral data reported from the present study do not provide direct evidence about the primary causes of bilateral interference. This should be further examined in future work using techniques that measure both brain activity, e.g., electroencephalography (EEG) or functional magnetic resonance imaging (fMRI), and muscle activity by electromyography (EMG), in order to provide additional insight in how inhibitory and excitatory interactions cause bilateral interference. Direct measurements of neural activity might help to disentangle the relative importance of cortical and spinal interactions causing bilateral interference.

The results illustrate that it is difficult to perform simultaneous non-isomorphic movements of the arms. Performance of a primary motor control task with the dominant arm suffers interference when a different motor task performed with the non-dominant arm is introduced and performed simultaneously. As hypothesized, this bilateral interference was found to be larger for proximal effectors compared to distal effectors, which is consistent with the differences in neuroanatomical and neurophysiological organization of proximal and distal effectors. These results pave the way for further research combining behavioral measurements with direct measurements of neural activity to clarify how bilateral communication influences bimanual performance in general, and potential differences between proximal and distal effectors.

In order to examine the hypothesis that bimanual interference affects proximal effectors more compared to distal effectors of the upper extremities, the current study used constrained circular movements of the non-dominant hand as the interference task. These movements were conducted with minimal temporal and spatial instruction, i.e., participants were in principle just asked to rotate the disk at a steady pace. In future research, it is interesting to examine bimanual interference when restricting the interference task to activate either proximal or distal muscles. It is likely to expect that specific restrictions of proximal and distal effectors could cause an even higher bimanual interference for proximal effectors, and less or equal bimanual interference for distal effectors, as a consequence of their differences in potential for bilateral communication. Additionally, proximal-distal control needs to be examined in *dual-task* conditions in which the bimanual interference task also includes precise temporal and spatial goals. This latter experimental paradigm could examine the hypothesis that dual-task performance is less stable in proximal compared to distal motor control by comparing and discriminating proximal and distal effectors bimanual stability, bimanual phase relations, jerk etc.

Furthermore, it would be interesting to study methods to overcome bimanual interference or enhance bimanual performance in general with specialized bimanual motor learning, and with the use of external utilities such as different types of exoskeletons.

## Conclusion

To conclude, the present study contributes to our understanding of bimanual interference and the association with the neuroanatomical and neurophysiological differences between proximal and distal effector systems. It was hypothesized that the neuroanatomical and neurophysiological differences between proximal and distal effectors would result in more pronounced bimanual interference in proximal compared to distal effectors. The results in the present study map the neuroanatomical and neurophysiological differences, and this was confirmed by the proximal-distal gradient in our results. Thus the study provides information about potential neuromuscular constraints on the motor control system of proximal and distal effectors, on bimanual interference in general and on the differences between bimanual interference observed in the proximal versus distal effector systems of the upper extremities in particular.

There seem to be an undesired bilateral interference for both proximal and distal muscles. The interference is higher in the case of proximal effectors compared distal effectors, and the results seem to map the neuroanatomical and neurophysiological differences for these effectors.

## Data Availability Statement

The raw data supporting the conclusions of this article will be made available by the authors, without undue reservation.

## Ethics Statement

The study was evaluated and approved by the Regional Ethical Committee and performed in accordance with the Declaration of Helsinki. The patients/participants provided their written informed consent to participate in this study.

## Author Contributions

All authors made contribution to the conception and design of the study, acquisition of data, analysis and interpretation of data, drafting the manuscript, revising the manuscript critically for important intellectual content, and approval of the final version of the manuscript to be published.

## Conflict of Interest

The authors declare that the research was conducted in the absence of any commercial or financial relationships that could be construed as a potential conflict of interest.

## Footnotes

1 http://core.ecu.edu/psyc/wuenschk/SPSS/SPSS-Programs.htm
